# Supplementary material for: A dynamic eco-evolutionary model predicts slow response of alpine plants to climate warming
Source: Nat Commun. 2017 May 5;8:15399. doi: 10.1038/ncomms15399 (PMC5424169; doi:10.1038/ncomms15399)
Supplement: Supplementary Information — Supplementary Figures, Supplementary Methods, Supplementary Tables, and Supplementary References [file ncomms15399-s1.pdf]

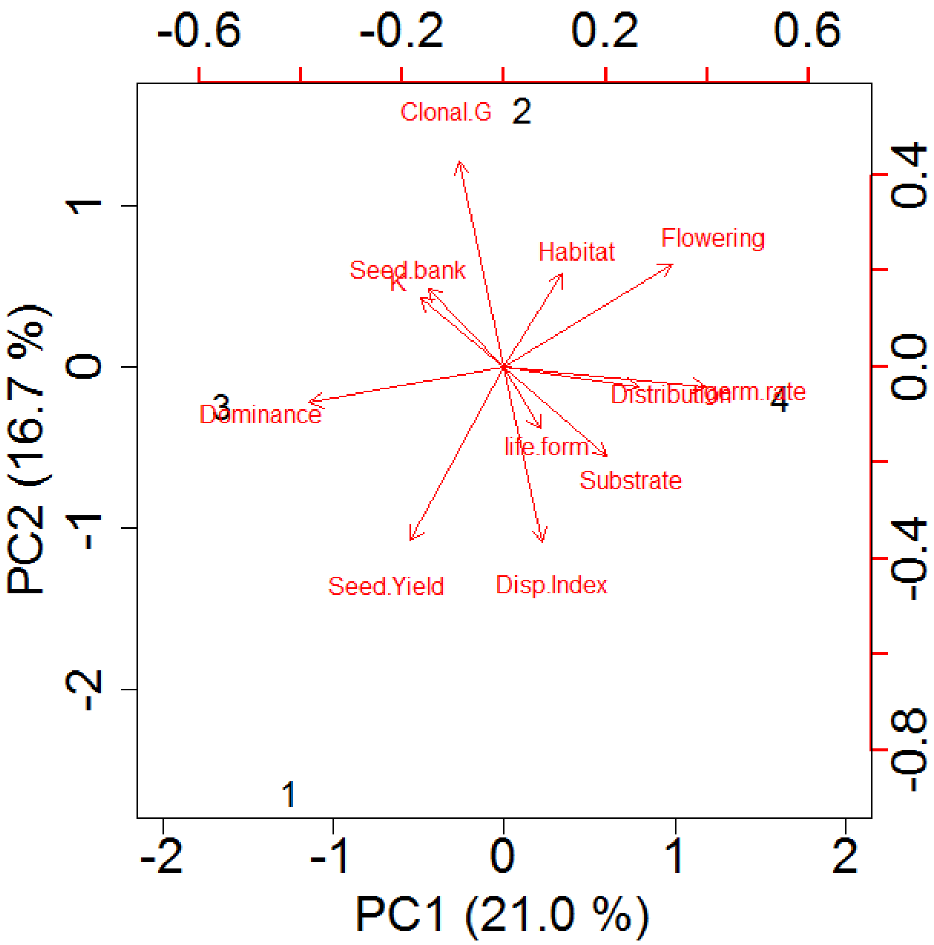

Supplementary Figure 1: Mapping of the selected species and factors along the first two principal component axes. Red arrows correspond to the factors. 1: *Campanula pulla*, 2: *Dianthus alpinus*, 3: *Festuca pseudodura*, 4: *Primula clusiana*.

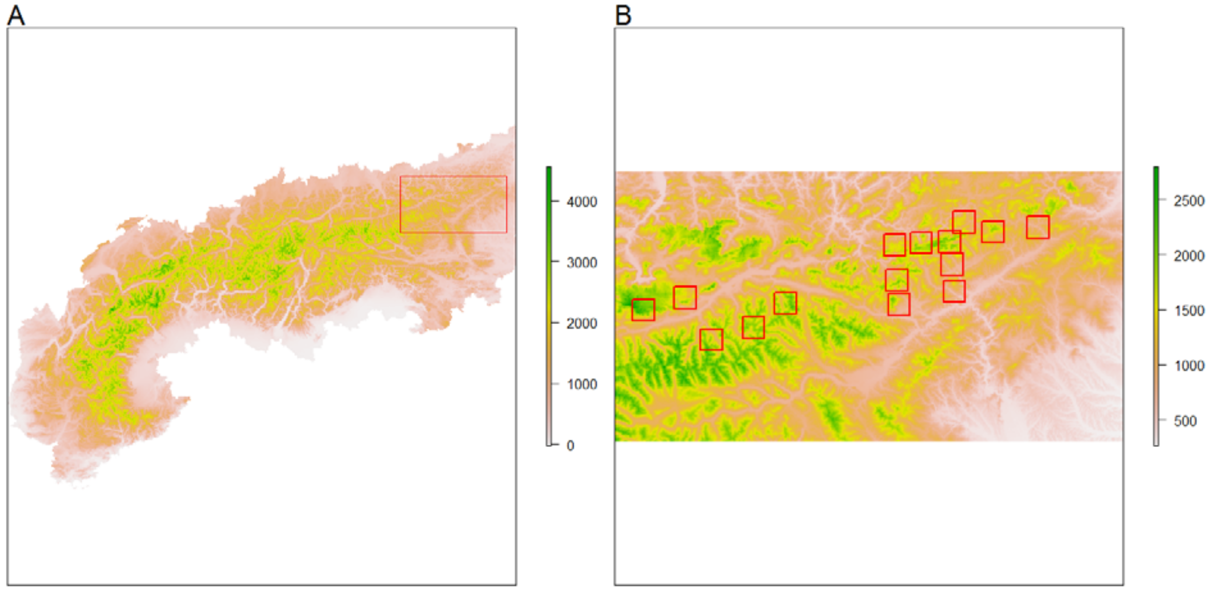

Supplementary Figure 2: A: Location of the study area within the Alps. B: Location of the studied grids (red boxes). The scale bar represents elevation above sea level in meters.

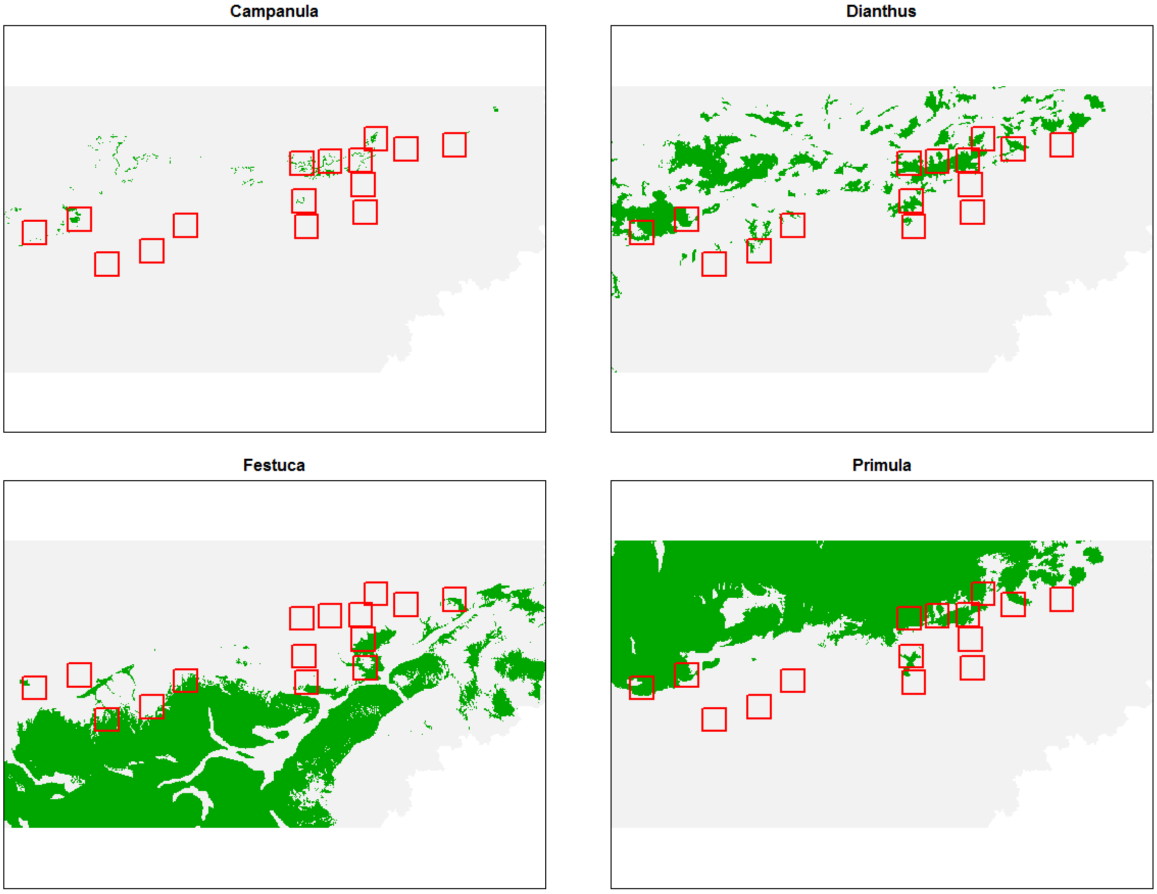

Supplementary Figure 3: Range (in green) of each species within the study area and location of the selected grids (red boxes). Species presence is derived from SENM predictions on climate (mean annual temperature and annual precipitation sum) averaged over the period 1950 -2000, and a soil variable (percentage of carbonates in bedrock material).

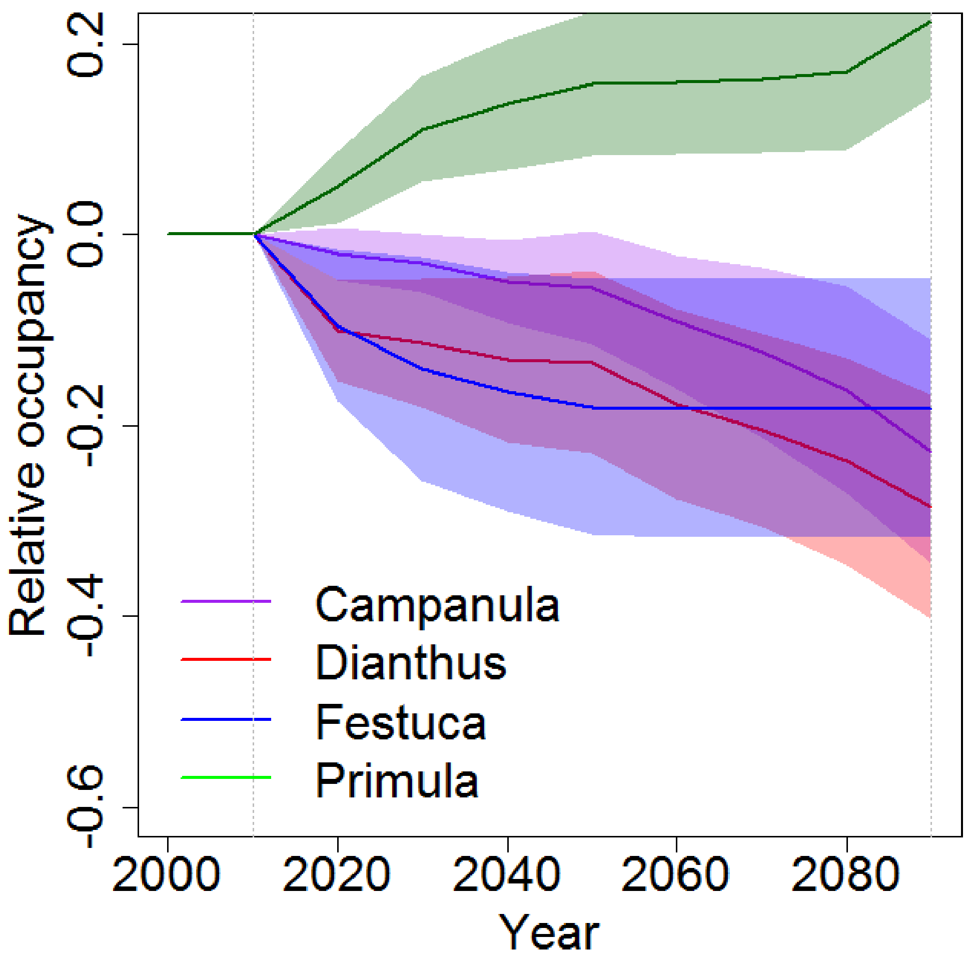

Supplementary Figure 4: Occupancy rate from the SENMs relative to initial occupancy (as calculated in Fig. 1) as a function of time and species. Lines: mean over grids and climatic scenarios, colored areas: standard deviations.

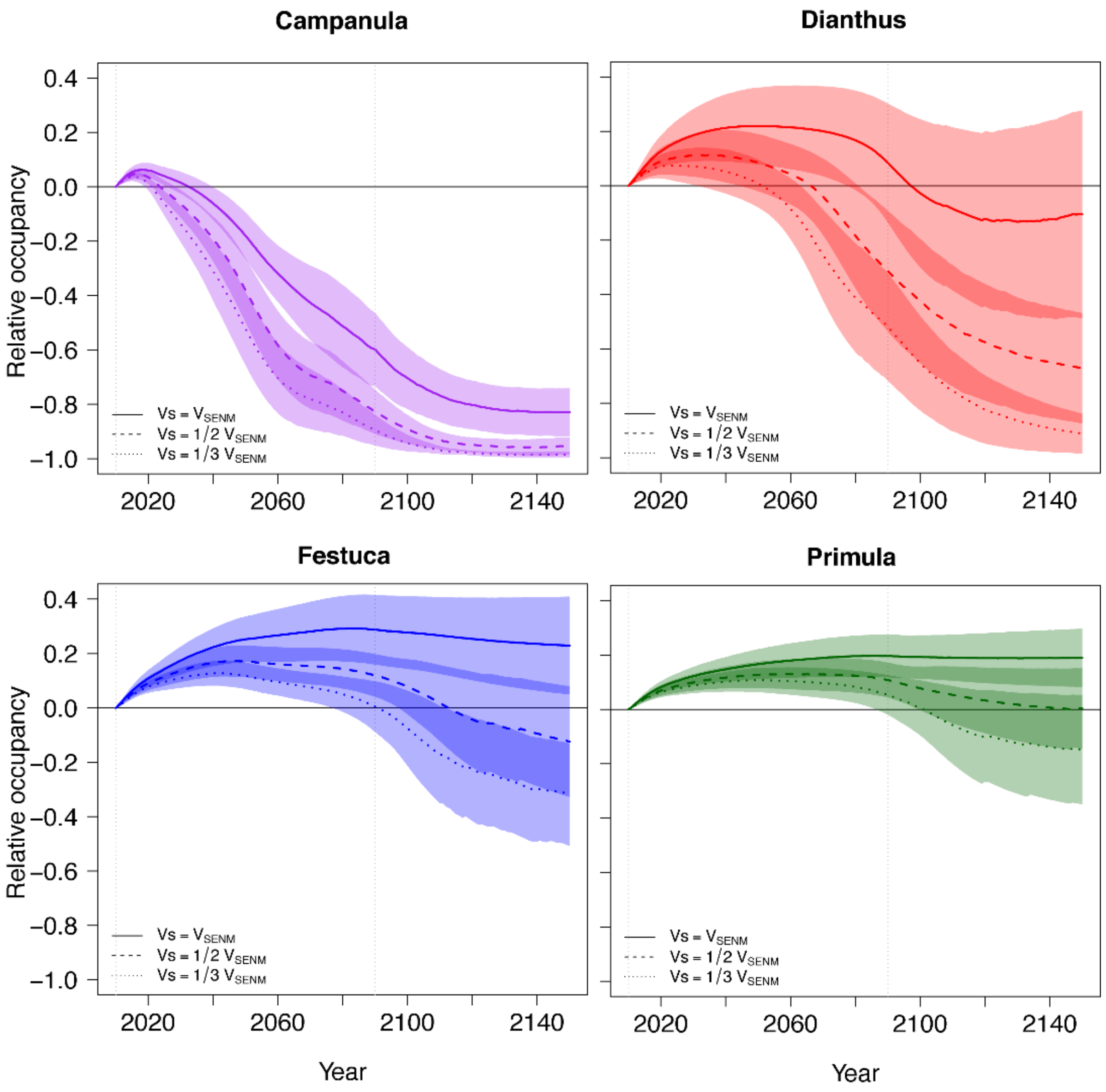

Supplementary Figure 5: Occupancy rate relative to occupancy in 2010 as a function of time and strength of natural selection. Occupancy is calculated as in Fig. 1. V_s is measured in proportion of the variance V_{SENM} of the current niche, i.e. the variance in the range of values of each environmental variable among the sites classified as suitable to the species by the SENMs. The lower V_s is, the stronger the stabilizing selection (equation 2). Lines: mean over all simulations, colored areas: standard deviation.

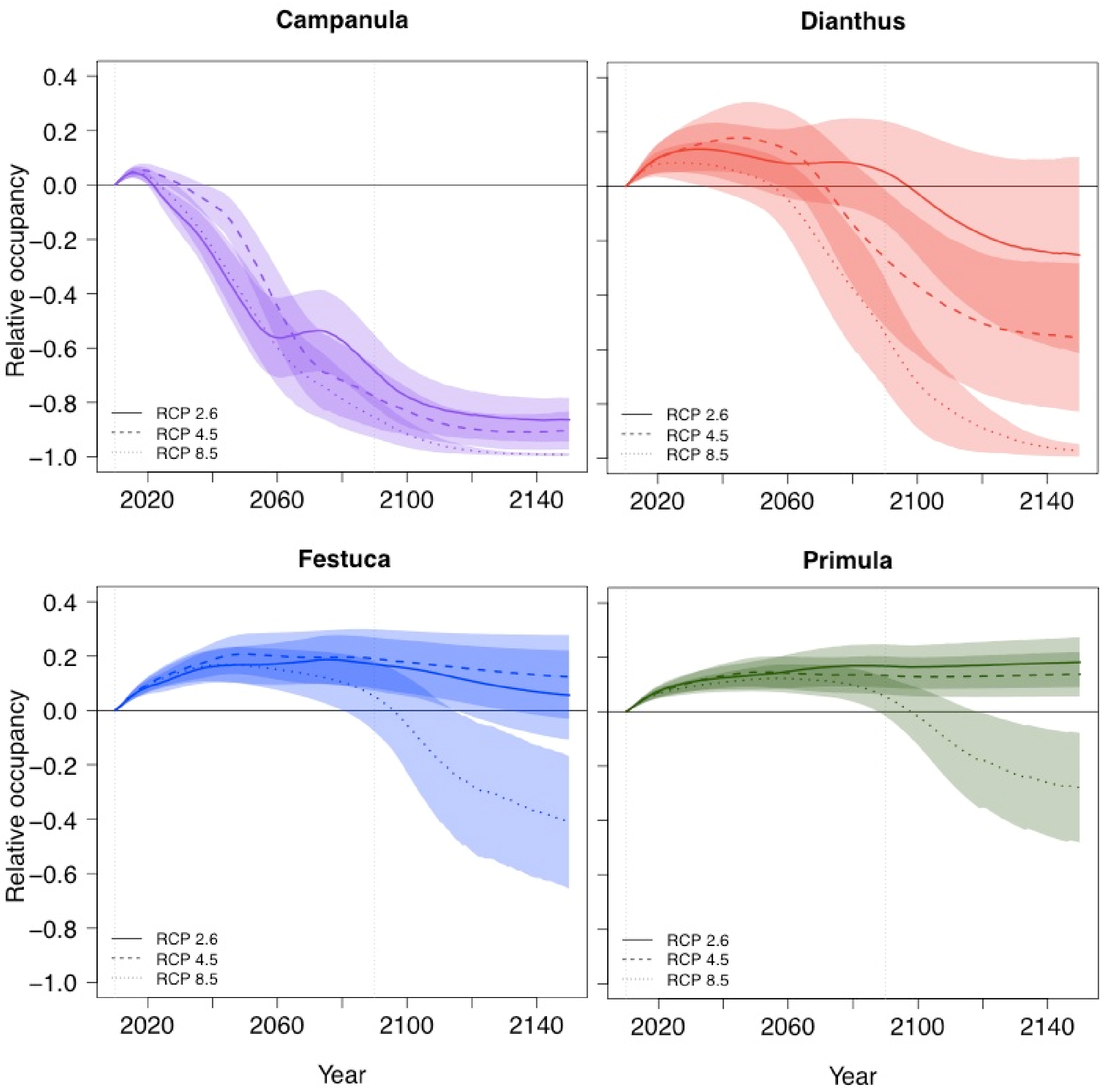

Supplementary Figure 6: Occupancy rate relative to occupancy in 2010 as a function of time and RCP scenarios. Occupancy is calculated as in Fig. 1. Lines: mean over all simulations, colored areas: standard deviation.

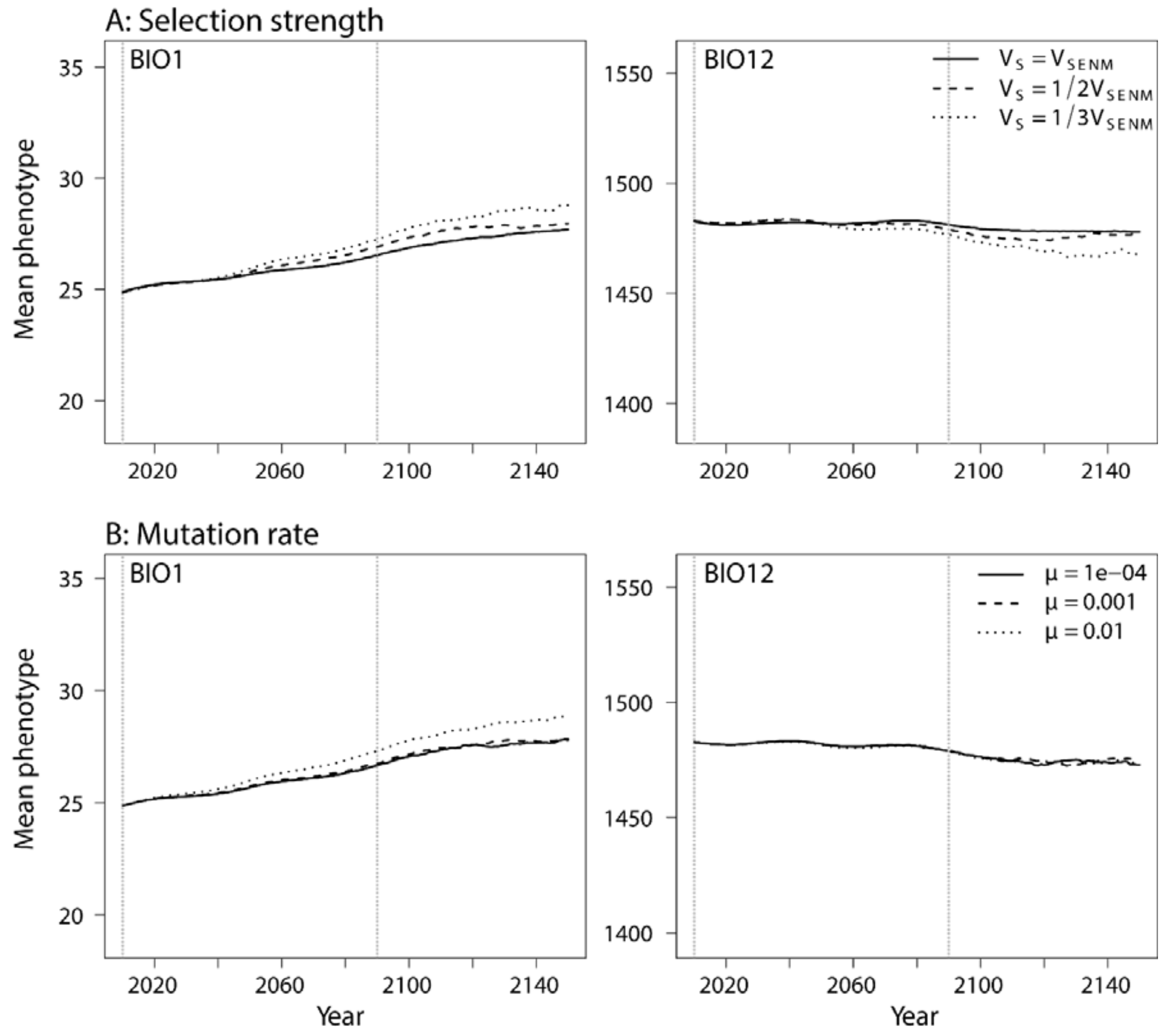

Supplementary Figure 7: Change in the mean trait values corresponding to the climatic variables Bio1(mean annual temperature) and Bio 12 (annual precipitation sum) as a function of time.

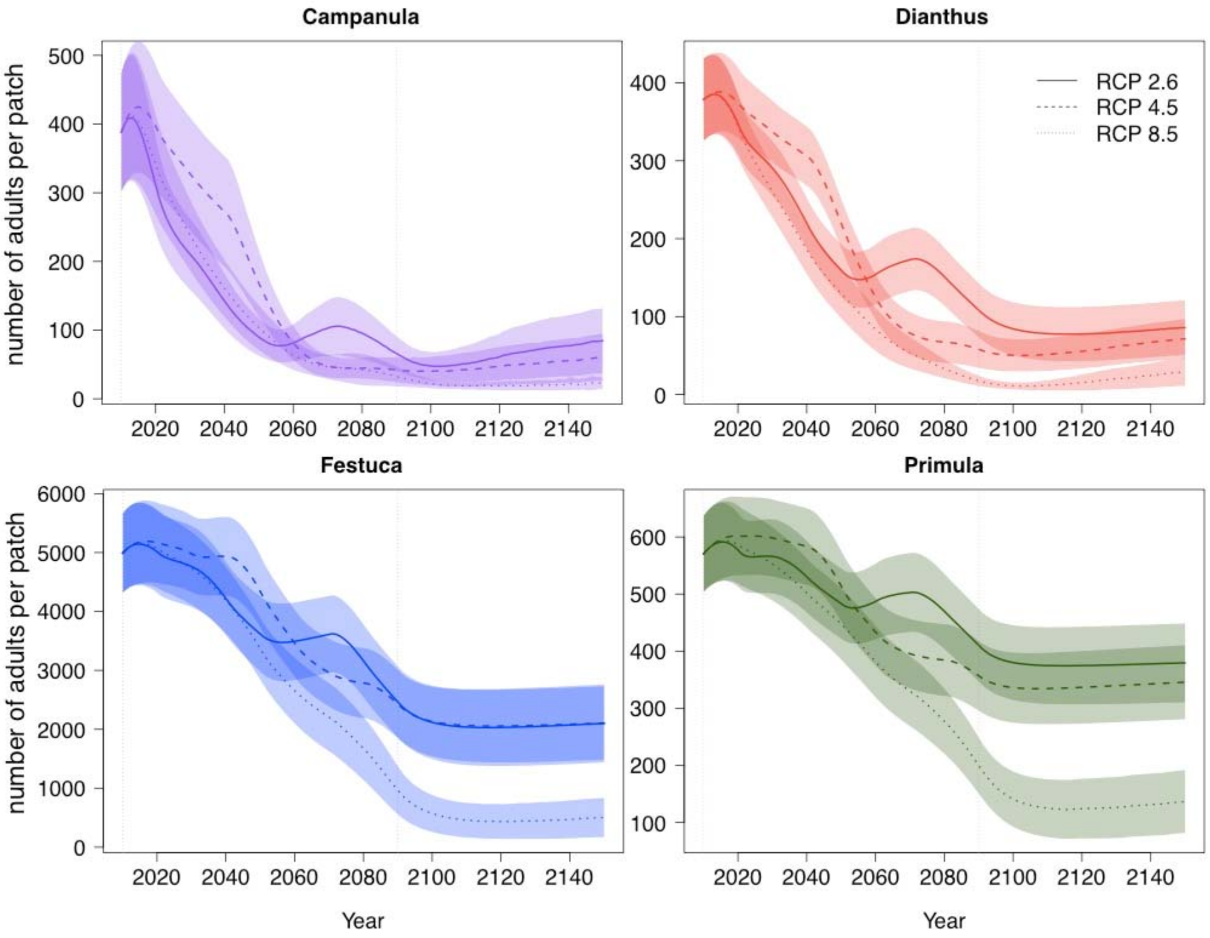

Supplementary Figure 8: Variation of population size of occupied sites between 2010 and 2150 for the three RCP scenarios. Lines are changes in population sizes averaged over all grids and evolutionary parameters in each species. Colored areas: standard deviation.

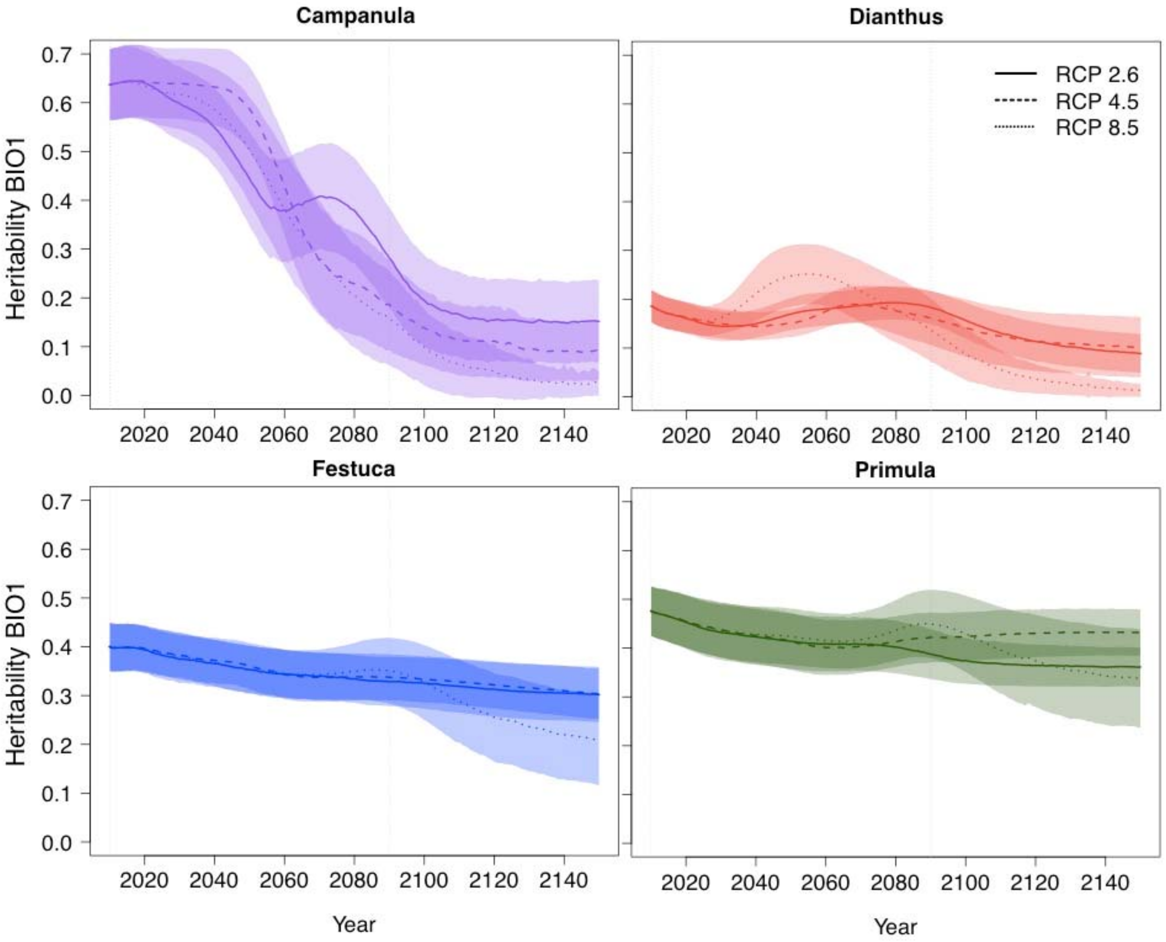

Supplementary Figure 9: Variation of trait (narrow-sense) heritability over time in each species for each RCP scenario for Bio 1 (mean annual temperature).

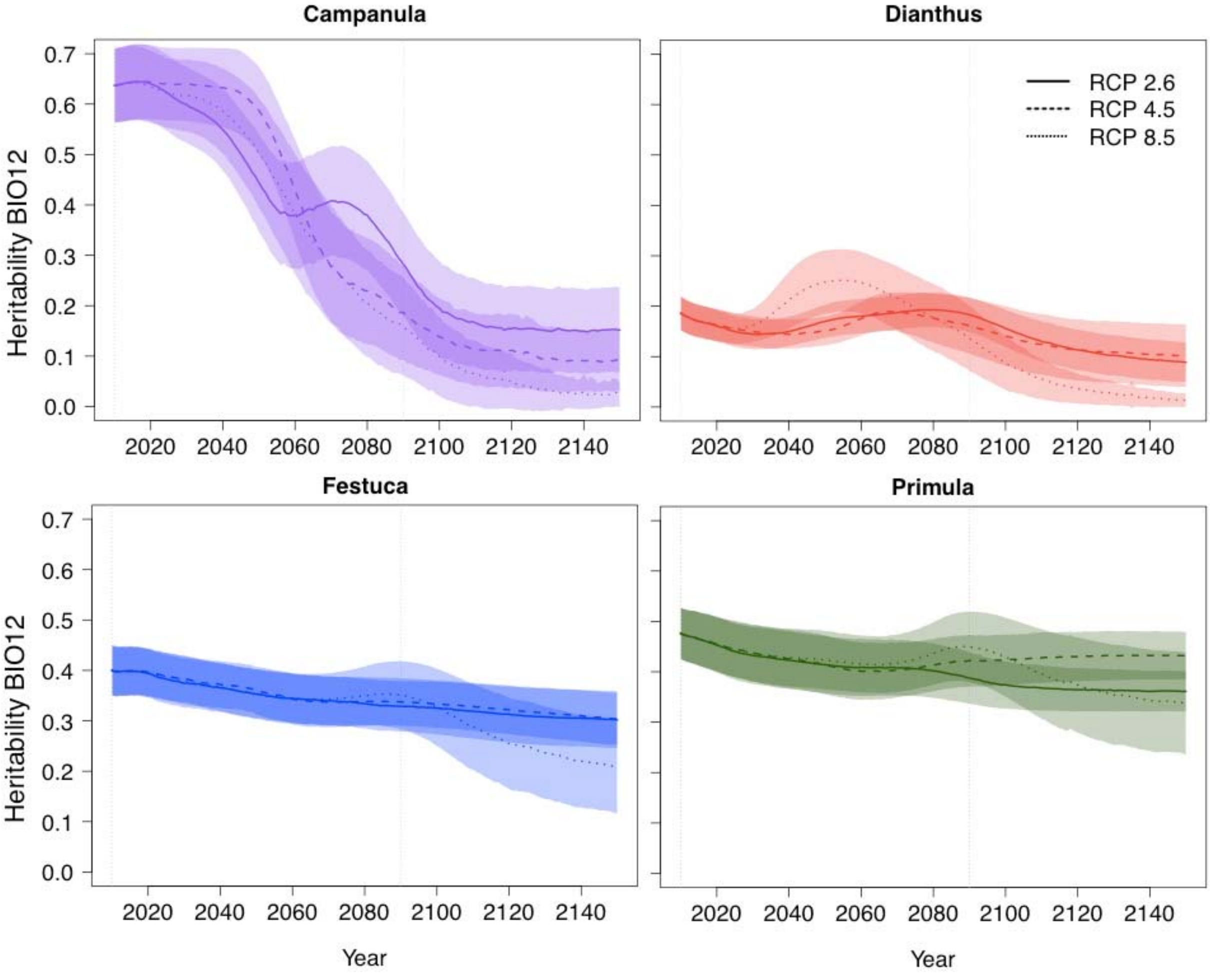

Supplementary Figure 10: Variation of trait (narrow sense) heritability over time in each species for each RCP scenario for Bio 12 (annual precipitation sum).

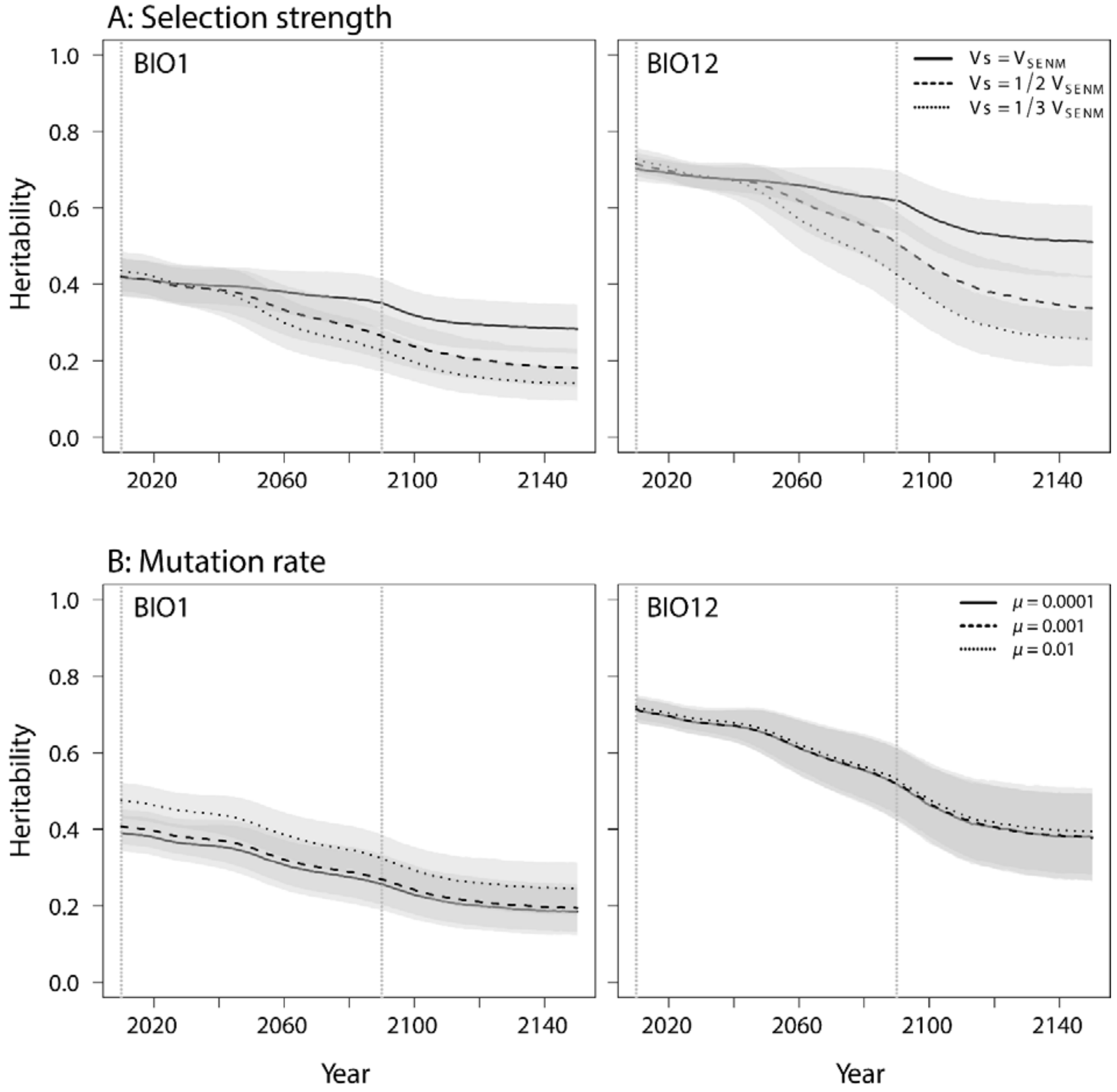

Supplementary Figure 11: Change in the mean heritability (narrow sense) for the traits corresponding to Bio 1 (mean annual temperature) and Bio 12 (annual precipitation sum) as a function of time.

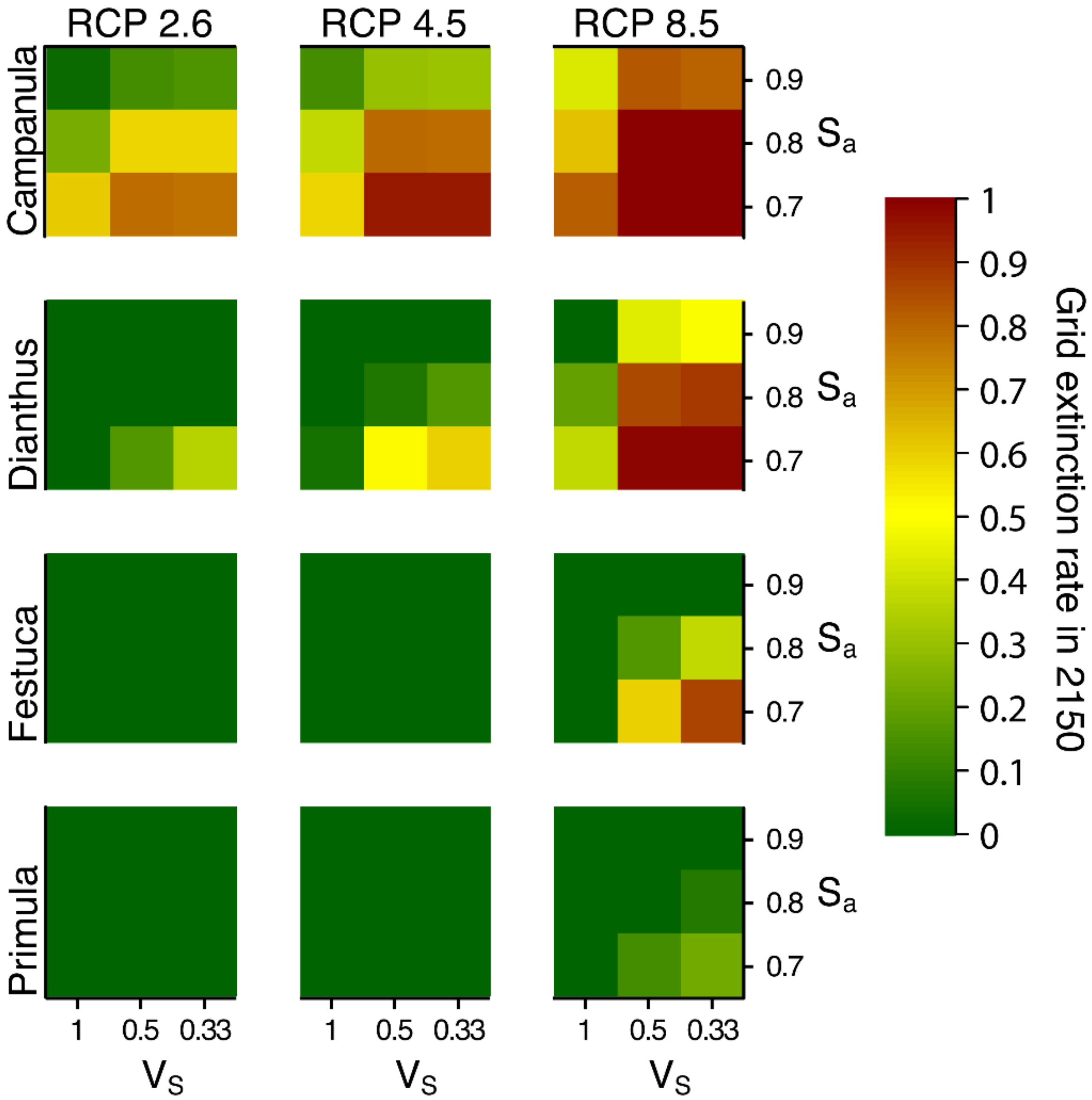

Supplementary Figure 12: Rate of grid extinction in 2150 (proportion of replicates that were extinct in 2150) split per species, selection strengths and adult survival rates. Each cell is the average over all grids and mutation rates.

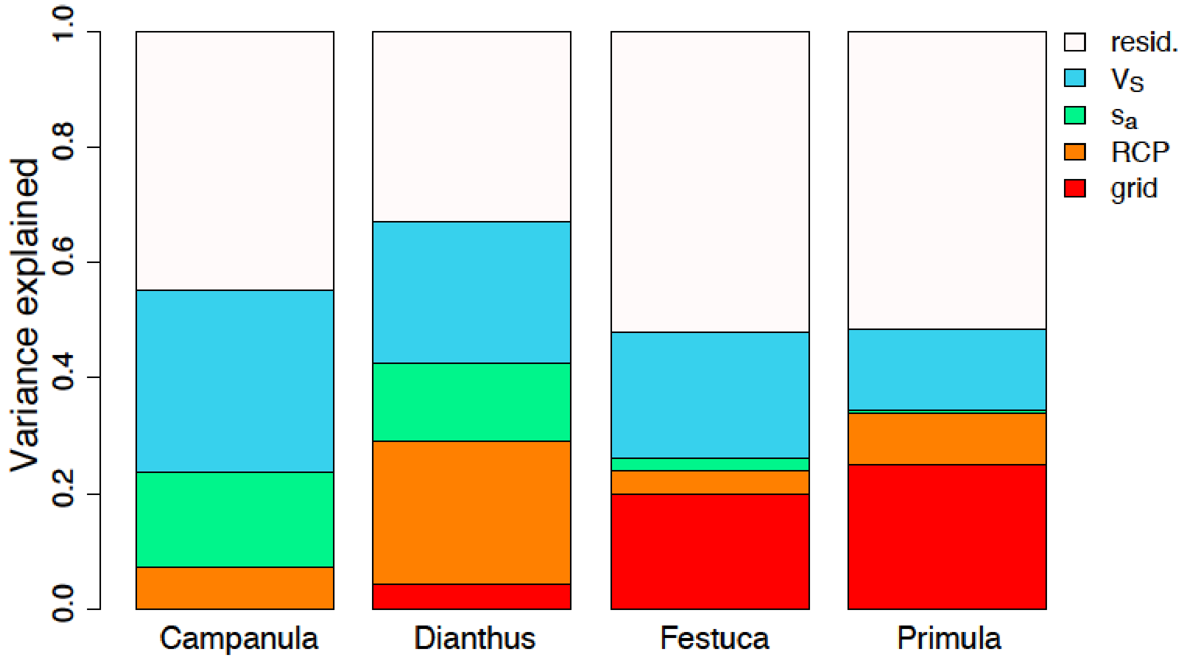

Supplementary Figure 13: Sensitivity analysis of the factors most affecting the variation in relative range size between 2010 and 2090, separately for each species. Only parameters explaining more than 2% of the variance in range size are illustrated. Residual variance (‘resid.’) is contributed by random variation among replicated simulations owing to stochasticity, and unaccounted factor interactions (all below 7% of variance explained, results not shown). Variation contributed by changing the mutation rate is too low to be represented. Variables included are: strength of selection (V_S), adult survival (s_a), climate change scenario (RCP), and simulated landscapes (grid).

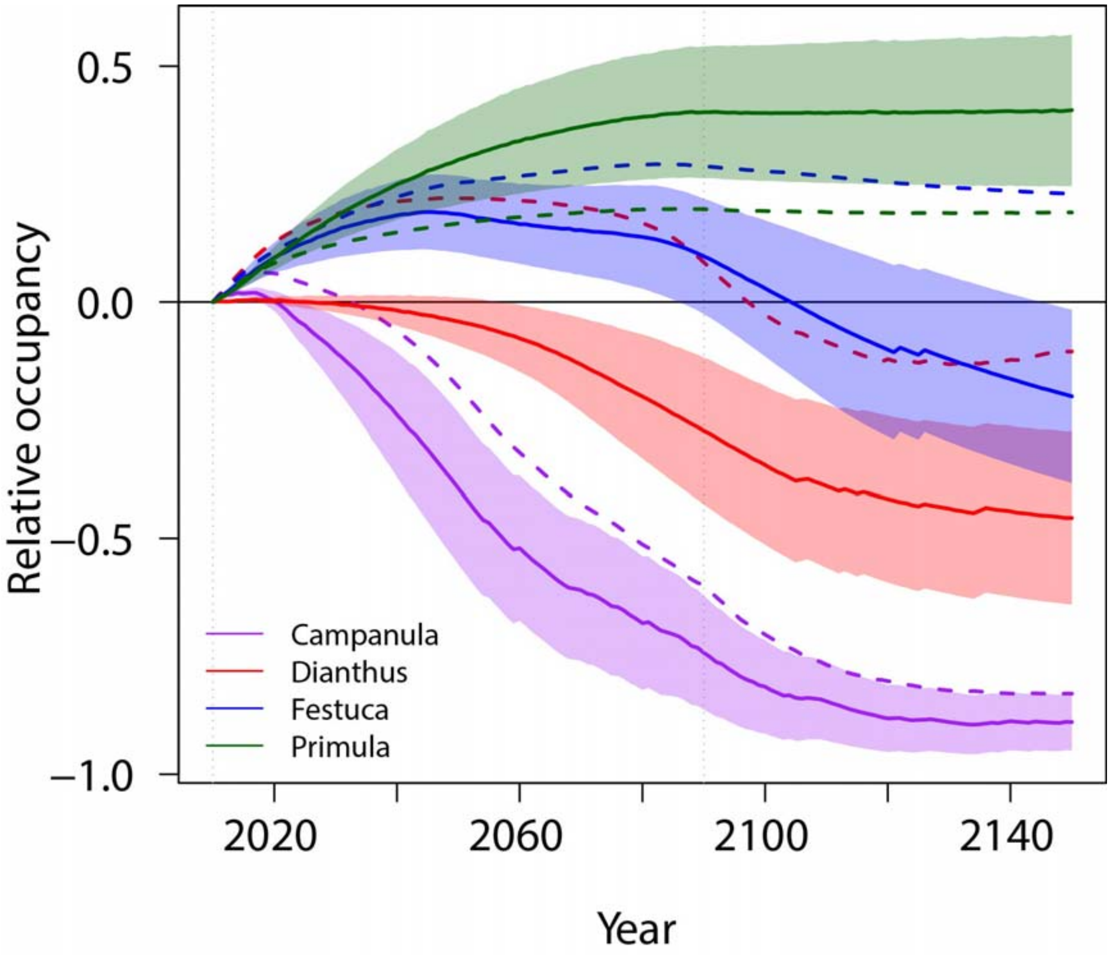

Supplementary Figure 14: Occupancy relative to the initial occupancy (as calculated in Fig. 1) as a function of time when considering a single globally adapted genotype. Line: mean over all grids, scenarios, species and replicates; dashed line: overall mean with local adaptation; colored areas: standard deviations. Vs $=V_{SENM}$.

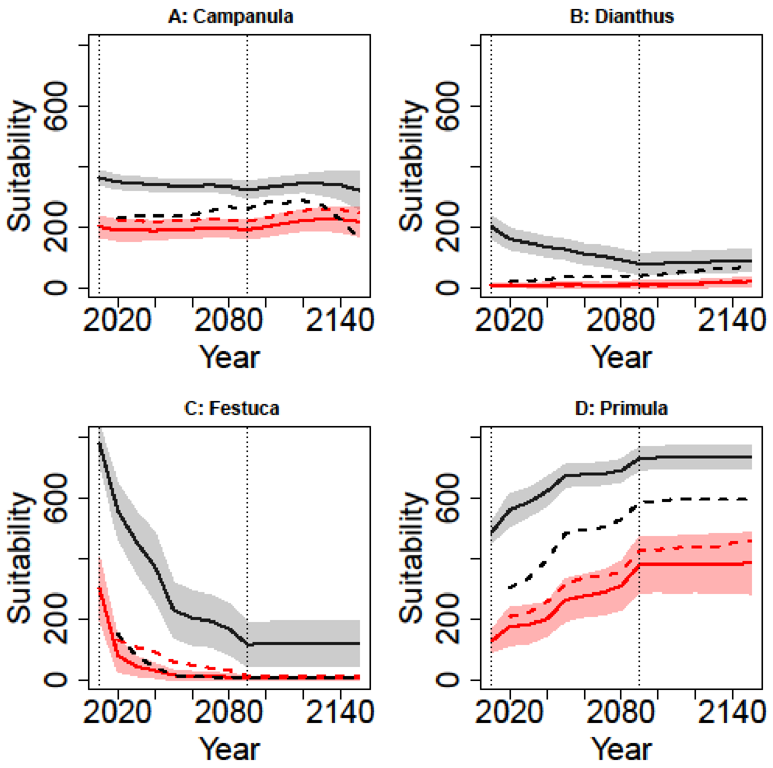

Supplementary Figure 15: Mean suitability (i.e. probability of presence x1000) predicted by the SENMs in the sites occupied (solid black line) and unoccupied in the DEEMs (solid red line). The dashed lines show the suitability in the sites colonized (black) and extinct (red) relative to the population state in 2010. Colored areas: standard deviations.

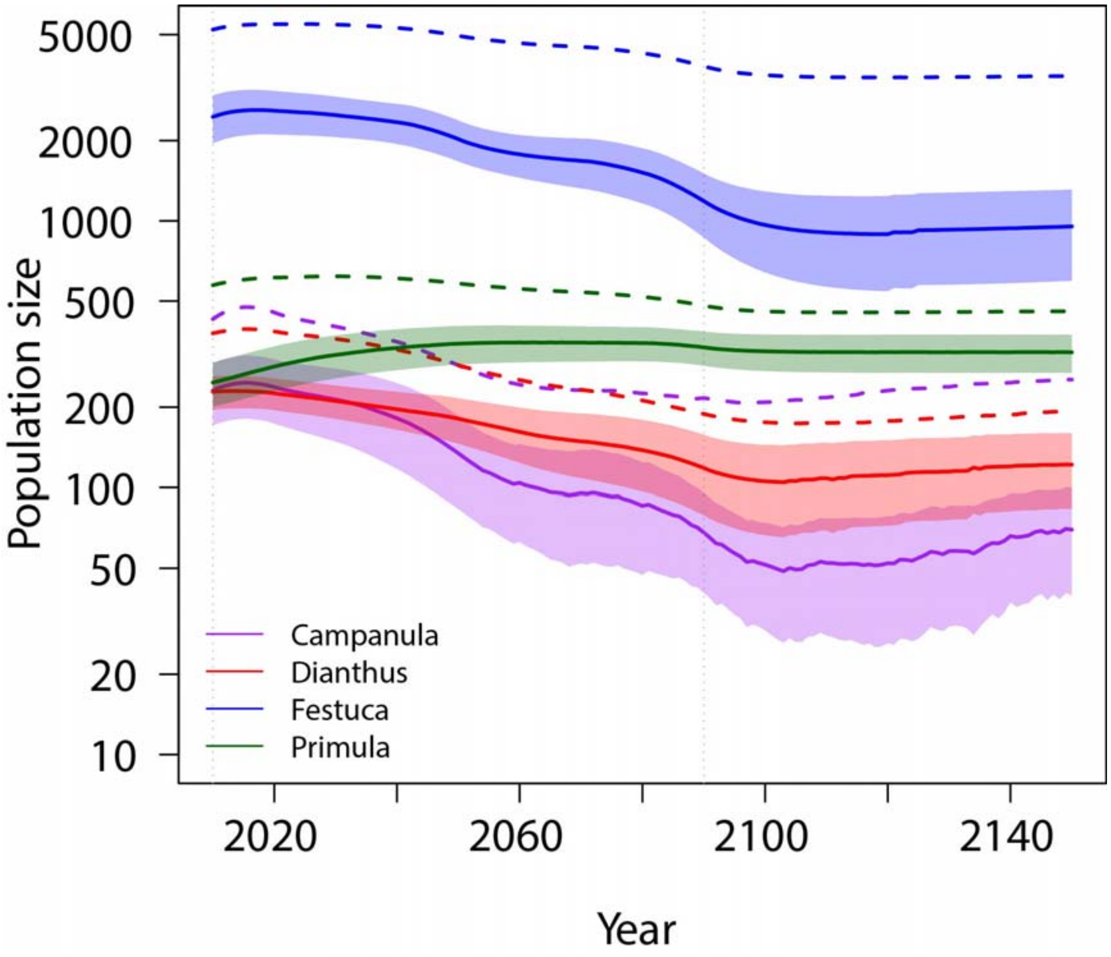

Supplementary Figure 16: Mean local population size as a function of time when considering a single globally adapted genotype. Lines: means over all grids, scenarios, species and replicates; dashed lines: overall means with local adaptation; colored areas: standard deviation. Vs =V_{SENM}.

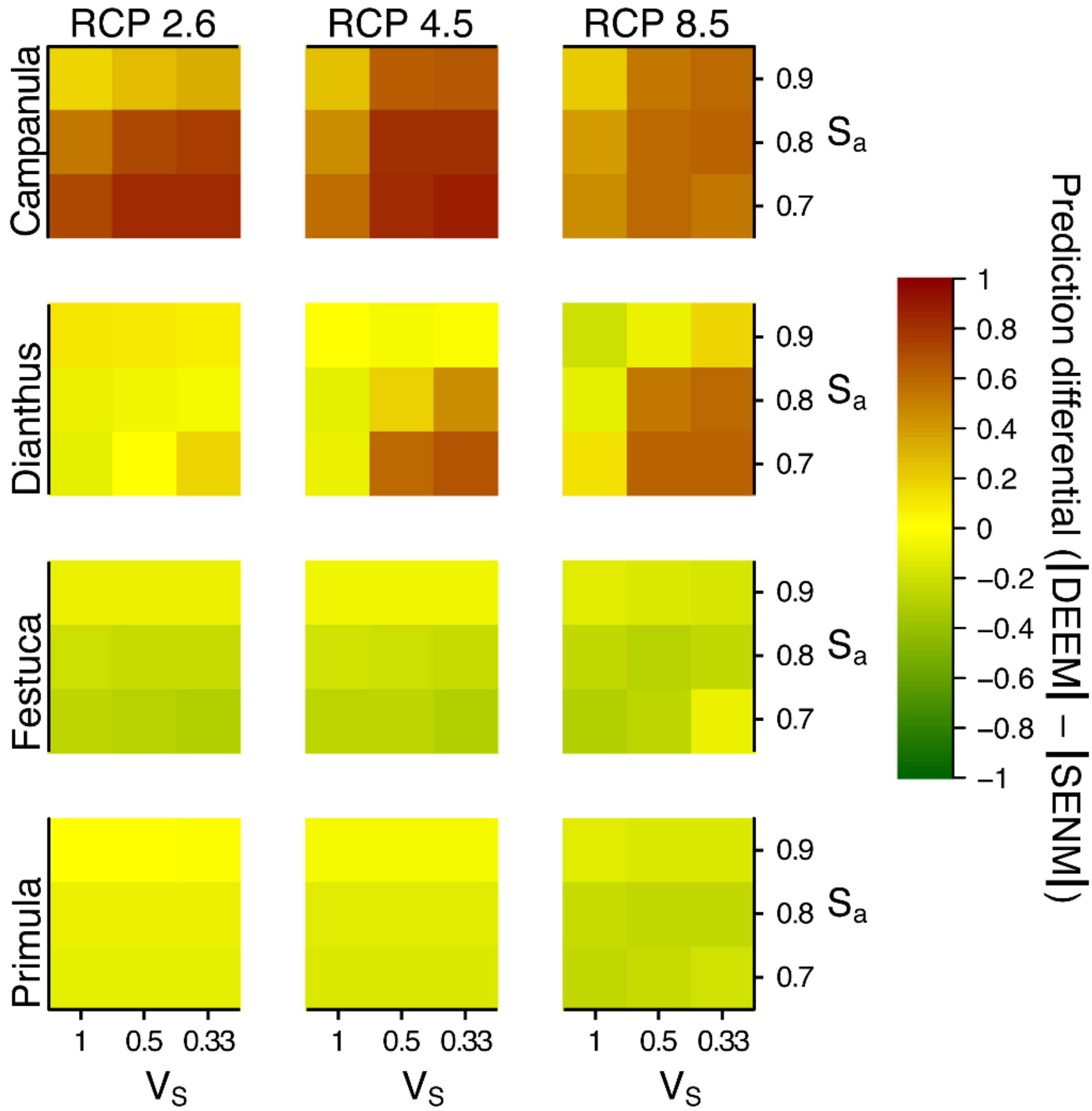

Supplementary Figure 17: Mean difference in relative occupancy in the year 2090 (as calculated in Figure 1) as predicted by DEEMs (over all grids, climate scenarios, and mutation rates) and SENMs (over all grids and climate scenarios), respectively. Positive values (warmer colours) indicate a larger absolute change of species' range in DEEMs compared to SENMs (i.e., does not inform on whether the predicted species' range expands or shrinks).

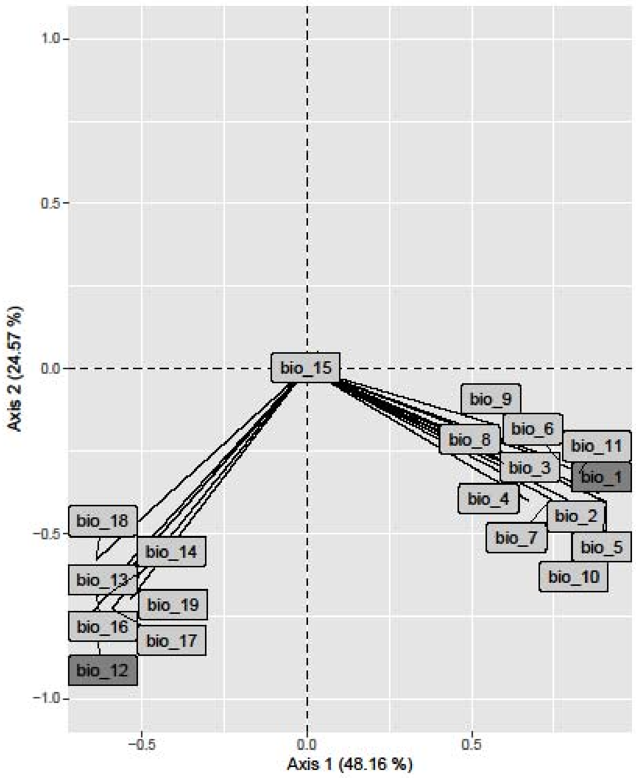

Supplementary Figure 18: PCA analysis on bioclimatic variables. Bioclimatic variables are from http://www.worldclim.org/bioclim. The bioclimatic variables used in this study (Bio 1 and Bio 12) are in dark-grey boxes.

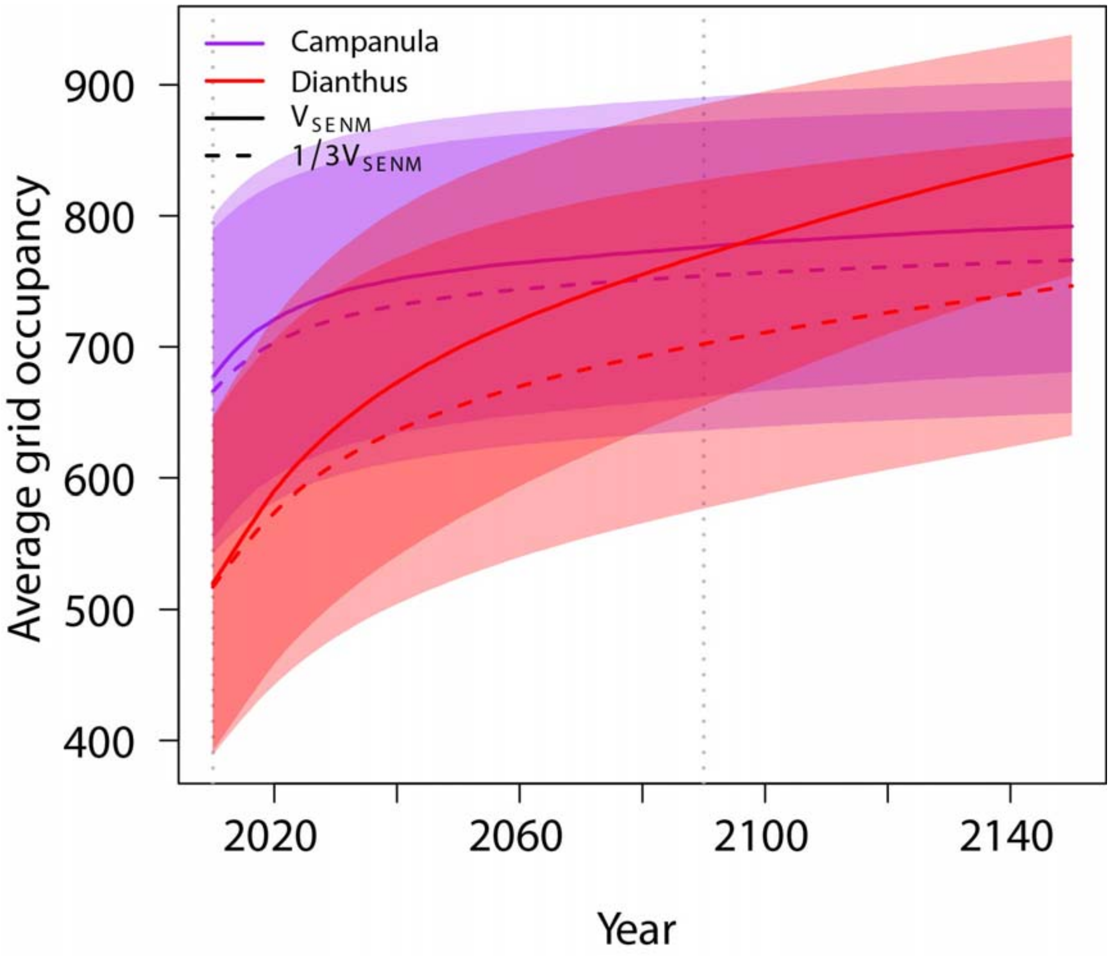

Supplementary Figure 19: Average grid occupancy (number of patches occupied per grid) of *Campanula* and *Dianthus* for two strengths of selection (100% and 33% of V_{SENM}) under the assumption of selection on adult fecundity (seed production).

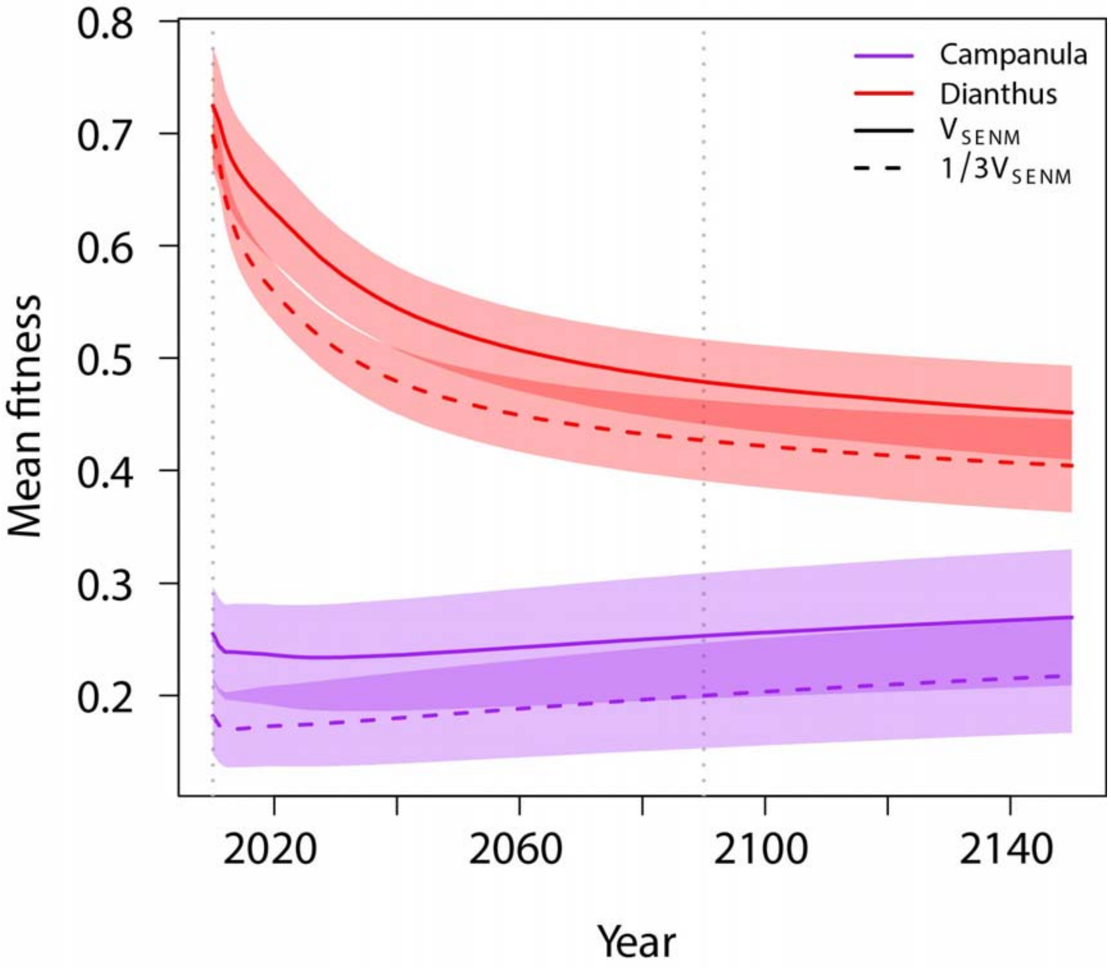

Supplementary Figure 20: Mean patch fitness (as given by equation 2) when selection is acting on adult fecundity in *Campanula* and *Dianthus* for two strengths of selection (100% and 33% of V_{SENM}).

SUPPLEMENTARY METHODS

Properties of the species' niche

Supplementary Table 1: Mean and variance (over all study landscapes = grids) of the environment in the sites that the SENMs predicted to be occupied by the four species. These statistics provide an overview of the current species niche in the parameter space defined by the three environmental variables used in this study (see Methods). We assumed that the variance in the environment occupied represents the lowest selection variance possible.

	Bio 1 (°C x 10)		Bio 12 (mm)		Carbonate (%)	
	Mean	Variance (V_{SENM})	Mean	Variance (V_{SENM})	Mean	Variance (V_{SENM})
Campanula pulla	26.9	15.2	1552.5	8179	63.3	37.9
Dianthus alpinus	19.3	110.4	1743.1	36591	75.6	756.2
Festuca pseudodura	36.3	459.2	978.5	36031	0.2	8.7
Primula clusiana	46.9	449.9	1617.3	61647	90.2	353.8

Supplementary Table 2: Environmental variances used to adjust heritability to ~0.3 for each trait and each selection variance.

	Bio 1			Bio 12		
	V_{SENM}	0.5 V_{SENM}	0.3 V_{SENM}	V_{SENM}	0.5 V_{SENM}	0.3 V_{SENM}
Campanula	2	1.5	1.3	45	37	28
Dianthus	3	2.7	2.4	54	39	31
Festuca	2.7	2.3	2	32	20	14
Primula	7.8	6.6	5.8	332	270	227

Current and Future Climate data

WorldClim (http://www.worldclim.org [1]) provides long-term monthly averages for precipitation and minimum, average and maximum temperature, and in addition a series of 19 bioclimatic variables directly derived from the monthly base maps at a resolution of 30 seconds (ca. 1 km).. We first downscaled the monthly base maps to a spatial resolution of 100m, in order to better represent topographic modification of micro-climate in our study area see [2]. In a second step we used these downscaled temperature and precipitation grids to generate maps of all 19 bioclimatic variables. In final step, we up-scaled these data to a 250m scale as a compromise between the area covered and the precision for local climate variability.

Future climate data were extracted from the Cordex data portal (http://cordexesg.dmi.dk/esgf-web-fe/live) and statistically downscaled from the original 11' resolution by (a) calculating differences ("deltas") in temperature and precipitation values between hindcasted historical (mean 1950 – 1999) and forecasted future climatic parameters at the original spatial resolution; (b) spatially interpolating these differences to a resolution of 100 x 100 m; (c) up-scaling the differences to the resolution of our bioclimatic base maps (250x250m); (d) adding these differences to the downscaled WorldClim maps of the same climatic variables [2,3]. Finally, we calculated 15-year means of these future time series for every tenth year (2013 - 2027, 2023 – 2037, …) and therefrom derived the 19 bioclimatic variables in decadal time steps.

We used three different IPCC5 scenarios from the new Representative Concentration Pathways-family (RCP 2.6, RCP 4.5, RCP 8.5) [4]. These scenarios reflect different radiative forcing trajectories for the 21st century relative to pre-industrial conditions: The RCP2.6 scenario assumes that radiative forcing peaks at ~ 3 W m^{-2} before 2100 and then declines and is therefore

referred to as mild scenario. In the intermediate scenario, RCP4.5, radiative forcing amounts to ~ 4.5 W m^{-2} at stabilization after 2100, while in the severe scenario, RCP8.5, radiative forcing continues to rise throughout the 21st century and reaches > 8.5 W m^{-2} in 2100 [4].

At the time of the downscaling, not all combinations of regional and global circulation models were available, so we chose one with a relatively smooth predicted time series for each RCP scenario, as compared to the other available options. Specifically, we used model runs from: (1) The Rossby Centre regional atmospheric model (RCA4), which was fed by the global circulation model EC-EARTH for the RCP 2.6 scenario generated by the Swedish Meteorological and Hydrological Institute (SMHI). (2) The HIRHAM5 model, which was fed by output from the global circulation model EC-EARTH for the RCP 4.5 scenario generated by the Danish Climate Centre (DMI). And (3), the Rossby Centre regional atmospheric model (RCA4), which was fed by output from the CNRM-CERFACS-CNRM-CM5 for the RCP 8.5 scenario generated by SMHI.

Geological data

A fine-scaled map of substrate units was available for the Austrian Alps [5] which we used to compute the area of calcareous substrates within grid cells.

Vegetation plot data

2386 localized plot data from subalpine and alpine non-forest vegetation of the Alps were compiled from published [6] and unpublished database (Supplementary Fig. 2). Data correspond to presence/ true-absence.

Life cycle: detailed demographic recursions

We give below the exact recursions corresponding to the simulations for *Dianthus*. The recursions for other species can be deduced from these recursions by adjusting for the longevity of seeds (equations 1-4) in the seedbank as given in Supplementary Table 3.

The recursions for *Dianthus* are:

$$N_{sb2}(t+1) = \big(N_a(t)s_a + N_{prea}(t)s_{prea}\big)Fs_{sb}(1-g) \quad 1$$

$$N_{sb3}(t+1) = N_{sb2}(t)s_{sb}(1-g) \quad 2$$

$$N_{sb4}(t+1) = N_{sb3}(t)s_{sb}(1-g) \quad 3$$

$$N_{sb5}(t+1) = N_{sb4}(t)s_{sb}(1-g) \quad 4$$

$$N_{sdl}(t+1) = \Big(\big(N_a(t)s_a + N_{prea}(t)s_{prea}\big)Fg + g\sum_{i=2}^{5} N_{sbi}(t) + C_g\big(N_a(t)s_a + N_{prea}(t)s_{prea}\big)\Big)\,c(t+1) \quad 5$$

$$N_{prea}(t+1) = N_{sdl}(t)s_{sdl} \quad 6$$

(Including selection, for individuals in patch *i* with phenotype **z** at time *t*: $N_{prea,i,\mathbf{z}}(t+1) = N_{sdl,i,\mathbf{z}}(t)s_{sdl}W_{i,t}(\mathbf{z})$, where $W_{i,t}(\mathbf{z})$) is given by equation 2 in Methods.)

$$N_a(t+1) = N_{prea}(t)s_{prea} + N_a(t)s_a\ , \quad 7$$

where $N_x(t)$ is the number of individuals in stage *x* at time *t* (*sbi*: seed having spent *i* - 1 years in the seedbank, *sdl*: seedlings, *prea*: preadults and *a*: adults), s_x the survival rate at stage *x*, *F* the fecundity of reproductive adults, *g* the germination rate, C_g the clonal fecundity and

$$c(t+1) = \frac{1}{1+kc\Big(\big(N_a(t)s_a+N_{prea}(t)s_{prea}\big)Fg+g\sum_{i=2}^{5}N_{sbi}(t)+N_a(t+1)+N_{prea}(t+1)+C_g\big(N_{sa}(t)s_a+N_{prea}(t)s_{prea}\big)\Big)}\ , \quad 8$$

is the competition coefficient (Beverton-Holt function), where *kc* is the competitive weight of an individual after germination. Since census occurs after seedbank survival and germination, seeds produced in year *t* either have germinated, died, or start their 2nd year in the seedbank.

Data about demographic rates were taken from Hülber et al. [7] Table S1 and the relevant numerical values are provided in Supplementary Table 3:

Supplementary Table 3: Demographic parameters for each species. Numerical values for demographic recursions as given in equations 1-8. Details on how these values are calculated are given below.

	Campanula	*Dianthus*	*Festuca*	*Primula*
Fecundity (*F*)	173*0.557=96	19*0.506=9.6	27*0.115=3.1	70*0.524=36.7
Seedbank survival rate (s_{sb})	NA	0.398	NA	0.63
Germination rate (*g*)	0.165	0.165	0.06	0.165
Seedling/pre-adult survival rate (s_{sdl}; s_{prea})	0.71	0.71	0.71	0.71
Adult survival rate (s_a)	0.7, 0.8, 0.9	0.7, 0.8, 0.9	0.7, 0.8, 0.9	0.7, 0.8, 0.9
Cloning rate (C_g)	0.5	2.625	3.5	1

Fecundity (= Seed Yield * Flowering frequency): We used the S values in Supplementary Table 3 from [7] (referred as H-2016 hereafter).

Seedbank survival rate: We kept the lowest estimation for the number of years a seed can survive in the seedbank (S values in H-2016). With this criterion, *Campanula* and *Festuca* have no seedbank. For *Dianthus* and *Primula* it is calculated with the rule that 1% of seeds in a given cohort survive to the estimated persistence in the seedbank, thus solving for $l_x = 0.01$, with $l_x = s_{sb}^{(x-1)}$ where s_{sb} is the yearly survival rate in the seedbank (assumed constant) and *x* the maximum years in the seedbank (5 for *Dianthus*, 7 for *Primula*). For *Primula*, we assumed that seed can stay a maximum of 7 years in the seedbank (instead of 10 as proposed in H-2016) to limit the memory required for the simulations (individuals in the seedbank need to be stored in memory) to a computationally manageable amount.

Germination rate: We used the S values in H-2016. These values correspond to measured germination rates times an estimate of the survival rate in the first year after germination from [8], which is here equal to 0.4.

Seedling/pre-adult survival rate: values from H-2016, correspond to the probability that an established seed survives to adulthood. Here we consider two post-establishment stages (seedlings and preadults, i.e. maturity is reached after 2 years). The values in Supplementary Table 3 satisfy $s_j^2 = 0.5$.

Adult survival rate: We performed a sensitivity analysis on this parameter because no precise estimates were available for the focal species. We considered 3 values (0.7, 0.8 and 0.9) according to estimated adult survival rates in species with similar ecology [9-11].

Clonal rate: Correspond to the cloning rates in H-2016 (median of the estimated values) minus 1 to remove the focal individual. We used the median of the range estimated as 1) the range of estimated values was wider for this parameter 2) the lowest estimated values were similar for most species while the maximal values were substantially different. Using the median of the estimated range allows to use a reasonable value, i.e. capturing species specificity, given the estimated ranges.

Density dependent regulation:

Supplementary Table 4: Competition coefficients *kc* used in the simulations. Values of the competition coefficient as used in equation 8 above.

	Adult Survival		
	0.7	0.8	0.9
Campanula	0.0014	0.002	0.0045
Dianthus	0.0037	0.0060	0.013
Festuca	0.00025	0.00041	0.0009
Primula	0.0028	0.0044	0.009

Dispersal

We used the dispersal kernels from Dullinger et al. [2]. All selected species are polychorous, so that the dispersal kernels are composed of a wind dispersal kernel (based on a mechanistic model) and an animal dispersal kernel (based on random-walk simulations and measured seed detachment rate from chamois fur). We used the high distance kernels as proposed by Dullinger et al. [2], where the animal dispersal kernels, with the more fat-tailed distribution, account for 5% of the total dispersal kernel.

Supplementary References

1 Hijmans, R. J., Cameron, S. E., Parra, J. L., Jones, P. G. & Jarvis, A. Very high resolution interpolated climate surfaces for global land areas. *International journal of climatology* **25**, 1965-1978 (2005).

2 Dullinger, S. *et al.* Extinction debt of high-mountain plants under twenty-first-century climate change. *Nature Climate Change* **2**, 619-622, doi:10.1038/nclimate1514 (2012).

3 Zimmermann, N. E. *et al.* Climatic extremes improve predictions of spatial patterns of tree species. *Proceedings of the National Academy of Sciences* **106**, 19723-19728 (2009).

4 Moss, R. H. *et al.* The next generation of scenarios for climate change research and assessment. *Nature* **463**, 747-756 (2010).

5 Bayer, I. & Pavlik, W. in *Proceedings of the 6th European Congress on Regional Geoscientific Cartography and Information Systems (EUREGEO), Earth and Man.* 109-111.

6 Willner, W., Berg, C. & Heiselmayer, P. Austrian vegetation database. *Biodivers Ecol* **4**, 333 (2012).

7 Hülber, K. *et al.* Uncertainty in predicting range dynamics of endemic alpine plants under climate warming. *Global change biology* **22**, 2608-2619 (2016).

8 Forbis, T. A. Seedling demography in an alpine ecosystem. *American Journal of Botany* **90**, 1197-1206, doi:10.3732/ajb.90.8.1197 (2003).

9 Weppler, T., Stoll, P. & Stöcklin, J. The relative importance of sexual and clonal reproduction for population growth in the long-lived alpine plant Geum reptans. *Journal of Ecology* **94**, 869-879 (2006).

10 Kuss, P., Rees, M., Ægisdóttir, H. H., Ellner, S. P. & Stöcklin, J. Evolutionary demography of long-lived monocarpic perennials: a time-lagged integral projection model. *Journal of Ecology* **96**, 821-832 (2008).

11 Gonzalo-Turpin, H. & Hazard, L. Local adaptation occurs along altitudinal gradient despite the existence of gene flow in the alpine plant species Festuca eskia. *Journal of Ecology* **97**, 742-751 (2009).
